# Severity of Coronary Artery Disease in Prediabetic Patients Undergoing Elective Coronary Angiography

**DOI:** 10.7759/cureus.7913

**Published:** 2020-05-01

**Authors:** Hafiz Muhammad Asif Zarif, Muhammad Shahzad Farid, Muhammad Shahid, Momin Rasheed Khan, Muhammad Shoaib Abid, Burhan Akhtar, Kashif A Hashmi, Muhammad Zakria, Aamna Khan

**Affiliations:** 1 Cardiology, Chaudhry Pervaiz Elahi Institute of Cardiology, Multan, PAK; 2 Family Medicine, Tehsil Headquarter Hospital Fort Munro, Dera Ghazi Khan, PAK; 3 Medicine, Bahauddin Zakariya University, Multan, PAK

**Keywords:** coronary artery disease, prediabetes

## Abstract

Introduction

Our objective was to determine the severity frequency of coronary artery disease (CAD) in prediabetes patients undergoing coronary angiography (CAG) in a catheterization laboratory.

Materials and methods

This descriptive comparative study was conducted on patients who were planned for elective CAG in the hospital from January 2019 to November 2019. The study includes patients age ≥40 years undergoing elective CAG with or without percutaneous coronary intervention/percutaneous transluminal coronary angioplasty. There were 458 patients (381 men and 77 women) in this study that were categorized into three groups on the basis on their glycated hemoglobin (HbA1c) levels: group I (n = 143) as non-diabetes, group II (n = 110) as prediabetes, and group III (n = 205) as diabetes. The severity of CAD was determined using the Gensini score.

Results

A total of 458 patients were included. Of these, 44.97% had hypertension; n = 36 (25.17%), n = 48 (43.63%), and n = 122 (59.51%) in group I, group II and group III, respectively (P = .0001). A total of 214 (46.72%) had a smoking history. There was a strong family history of CAD in group II (n = 29, 26.36%) and group III (n = 43, 20.98%). Group II and group III patients had a higher extension of CAD than group I (P = .01). Group II (n = 27, 41.54) and group III (n = 65, 50.39%) had a higher frequency of deployment of two stents compared to group I.

Conclusion

Coronary artery atherosclerosis disease increases parallel to the HbA1c severity and smoking. The present study emphasizes prediabetes as an independent risk factor for CAD.

## Introduction

Diabetes is an unusual level of blood glucose that damages the body’s ability to process blood sugar. Prediabetes is an intermediate condition between diabetes and non-diabetes glucose values. Prediabetes patients are more prone to type 2 diabetes (T2D) within three to five years [1]. In the United States, one in three individuals has prediabetes (an estimated 33.9%) [2]. There is a prevalence rate of 7.9% around the world and 10.91% in Pakistan [3,4]. Individuals with prediabetes may have a 20% higher chance of cardiovascular disease and atherosclerosis risk compared to non-diabetic individuals [5]. Prediabetes causes diffuse lesions and creates tiny blood vessels that make unstable plaques in coronary arteries [6,7]. Early diagnosis and control of prediabetes are necessary to avoid T2D and vascular complications.

In 2008, the American Diabetes Association and the International Diabetes Federation formed a committee to review and diagnose T2D via a convenient and easy method. The committee recommended using glycated hemoglobin (HbA1c) level to provide an accurate diagnosis of T2D. HbA1c measurement provides a better diagnosis than fasting blood glucose and oral glucose tolerance test [8]. The present study was conducted to observe and compare the severity of coronary artery disease (CAD) in prediabetes patients with those of healthy individuals and T2D patients.

## Materials and methods

This was a descriptive comparative study, conducted at the Chaudhry Pervaiz Elahi Institute of Cardiology of Multan, Pakistan. Patients with chest pain and undergoing elective coronary angiography (CAG) in a cardiac catheterization lab (CCL) from January 2019 to November 2019 were included. The study included patients age ≥40 years undergoing elective CAG with or without percutaneous coronary intervention (PCI)/percutaneous transluminal coronary angioplasty (PTCA). No specific exclusion criteria were selected. The study was approved by the institutional ethical committee and research body. Written and informed consent was obtained from all patients.

The patient’s risk factors for CAD included their smoking history, diabetes status, hypertension (HTN), and the presence of a family history of CAD. Additionally, serum creatinine levels were recorded based on HbA1c base levels [9]. Based on their HbA1c level, patients were assigned into one of three groups: (i) non-diabetes (group I: HbA1c < 5.7%), (ii) prediabetes (group II: HbA1c = 5.7% to 6.4%), or (iii) T2D (group III: HbA1c > 6.4%).

The atherosclerotic burden on CAD vessels was assessed by the Gensini score (Table 1). The number of stents used was recorded group-wise. Other blood parameters such as plasma glucose, HbA1c, creatinine kinase, electrolytes, and blood count were analyzed in the central laboratory of the hospital.

**Table 1:** Gensini severity score of atherosclerotic measure

Reduction of coronary diameter	Severity score
1% to 25%	1
25% to 50%	2
51% to 75%	4
76% to 90%	8
91% to 99%	16
100%	32

CAG was performed using the Judkins method with a puncture of either radial artery or femoral artery. CAD extension was defined as shown in Table 2. CAG results were assessed by two individual cardiologists. An in-house Philips software system calculated the percentage of coronary stenosis in CCL.

**Table 2:** Baseline characteristics and risk factors for patients enrolled BUN, blood urea nitrogen; CABG, coronary artery bypass grafting; CAD, coronary artery disease; CHD, coronary heart disease; HDL-C, high-density lipoprotein cholesterol; LDL-C, low-density lipoprotein cholesterol; LVEF, left ventricular ejection fraction; MI, myocardial infarction; PCI, percutaneous coronary intervention; STEMI, ST-elevation myocardial infarction; ST, S wave T wave segment; TC, total cholesterol; TG, triglycerides

Characteristics	Group I (n = 143)	Group II (n = 110)	Group III (n = 205)	Test value	P value
Age ± SD (years)	53.42 ± 4.36	54.26 ± 8.26	54.39 ±7.37	0.915	.40
Men, n (%)	124 (86.71%)	95 (86.36%)	162 (79.02)	4.6	.1
HbA1c, mean ± SD	5.32 ± 0.32	5.91 ± 0.16	8.64 ± 0.85	1.49	< .001
Hypertension, n (%)	36 (25.17%)	48 (43.63%)	122 (59.51%)	30.5	.001
Smoking, n (%)	49 (34.26%)	62 (56.36%)	103 (50.24%)	14.04	.17
Dyslipidemia, n (%)	112 (78.32%)	97 (88.18%)	189 (92.20%)	14.45	.007
Prior MI, n (%)	23 (16.8%)	28 (25.45%)	37 (18.05%)	3.84	.14
Prior PCI, n (%)	18 (12.59%)	23 (20.91%)	46 (22.44%)	5.6	.06
Prior CABG, n (%)	8 (5.59%)	2 (1.82%)	14 (6.83%)	3.67	.15
Family history of CAD, n (%)	12 (8.39%)	29 (26.36%)	43 (20.98%)	15.12	.005
Type of CHD					
Stable angina, n (%)	90 (62.93%)	60 (54.3%)	107 (52.0%)	4.09	.12
Unstable angina, n (%)	31 (21.67%)	24 (21.5%)	50 (24.2%)	0.45	.79
Non-STEMI, n (%)	13 (9.09%)	11 (10.1%)	27 (13.2%)	1.6	.44
STEMI, n (%)	10 (6.99%)	17 (15.1%)	22 (10.6%)	4.6	.09
ST-depression (> 1 mm), n (%)	17 (11.88%)	15 (13.2%)	33 (16.2%)	1.26	.53
LVEF (%) ± SD	55.24 ± 4.2	51.62 ± 6.4	47.87 ± 7.2	31.72	< .001
Lipid profile					
TG, mmol/L	1.8 ± 1.1	1.7 ± 1.0	1.7 ± 1.1	0.42	.65
TC, mmol/L	4.3 ± 1.2	4.3 ± 1.1	4.4 ± 1.1	0.44	.64
HDL-C, mmol/L	1.1 ± 0.2	1.0 ± 0.3	1.1 ± 0.2	8.04	.001
LDL-C, mmol/L	2.3 ± 0.8	2.4 ± 0.9	2.6 ± 0.9	5.33	.005
BUN, mmol/L	5.8 ± 2.2	5.6 ± 2.0	5.8 ± 2.4	0.33	.70
Creatinine, µmol/L	74.2 ± 15.3	77.3 ± 12.6	75.2 ± 9.2	2.05	.13

Study data were analyzed using Statistical Package for the Social Sciences (SPSS) for Windows, Version 23.0. (IBM Corp., Armonk, NY). A Student’s t-test was used to assess the difference between two study means, and a Chi-square test was applied to compare two study variables. We applied analysis of variance testing to determine the significance of the parametric variable among the groups.

## Results

Patient demographics and baseline clinical values are presented in Table 2. Group I contained 143 patients (124 men, 86.71%; 19 women, 13.28%). Group II contained 110 patients (95 men, 86.36%; 15 women, 13.64%). Group III contained 205 patients (162 men, 79.02%; 43 women, 20.97%; P = .40). A total of 206 (44.97%) patients had HTN; n = 36 (25.17%), n = 48 (43.63%), and n = 122 (59.51%) in group I, group II and group III, respectively (P = .0001). A total of 214 (46.72%) had a smoking history (P = .17). Dyslipidemia was diagnosed in group I (n = 112, 78.32%), group II (n = 97, 88.18%), and group III (n = 189, 92.20%), respectively (P = .007). There was a strong family history of CAD in group II (n = 29, 26.36%) and group III (n = 43, 20.98%) compared to group I (n = 12, 8.39%; P = .005). High-density lipoprotein cholesterol values were decreased, and low-density lipoprotein cholesterol values were elevated in all three groups (P = .001 and P = .005, respectively).

Interventional data are shown in Table 3. Group II (n = 27, 41.54) and group III (n = 65, 50.39%) had a higher frequency of deployment of two stents compared to group I (n = 25, 33.33%, P = .17). Group II had more coronary atherosclerotic complex anatomy with a higher Gensini score (28.0 ± 6.0, P < .001) than group I (20.0 ± 6.0) and group III (22.0 ± 8.0).

**Table 3:** Comparison of interventional data between the groups BMS, bare metal stent; CABG, coronary artery bypass grafting; CAG, coronary angiography; DES, drug-eluting stent; DVD, double vessel disease; LMCA, left main coronary artery; PCI, percutaneous coronary intervention; PTCA, percutaneous transluminal coronary angioplasty; SVD, single vessel disease; TVD; triple vessel disease

Characteristics		Group I (n = 143)	Group II (n = 110)	Group III (n = 205)	P value
Procedure, n (%)	CAG	65 (45.45%)	43 (39.09%)	72 (35.12%)	.42
	CAG + PCI	75 (52.45%)	65 (59.09%)	129 (62.93%)	
	CA + PTCA	3 (2.1%)	2 (1.82%)	4 (1.95%)	
Diseased vessels extent, n (%)	No CAD	28 (19.58%)	13 (11.82%)	25 (12.2%)	.01
	SVD	58 (40.56%)	33 (30%)	51 (24.88%)	
	DVD	29 (20.28%)	31 (28.18%)	67 (32.68%)	
	TVD	18 (12.59%)	21 (19.09%)	43 (20.98%)	
	LMCA	10 (6.99%)	12 (10.91%)	19 (9.27%)	
Gensini score	Mean ± SD	20.0 ± 6.0	28.0 ± 6.0	22.0 ± 8.0	< .001
Type of stent, n (%)	BMS	2 (2.67%)	2 (3.08%)	3 (2.33%)	.95
	DES	73 (97.33%)	63 (96.92%)	126 (97.67%)	
Quantity of stent, n (%)	1	46 (61.33%)	34 (52.31%)	56 (43.41%)	.17
	2	25 (33.33%)	27 (41.54%)	65 (50.39%)	
	3	4 (5.33%)	4 (6.15%)	8 (6.2%)	
Referred for CABG	n (%)	37 (25.87%)	30 (27.27%)	47 (22.93%)	.66

## Discussion

We assessed the incidence of atherosclerosis CAD and its burden in prediabetes and its impact on coronary vessels and CAG outcomes. In our study, 110 (24.01%) patients had prediabetes; the prevalence of men was high in all groups, while in other studies, women were predominant in the diabetes group [10,11]. The frequency of prediabetes with smoking and CAG + PCI/PTCA is higher in the prediabetes group, similar to the other studies conducted by Muhammed et al. [10]. Our study results found no statistical significance in smoking among the groups. However, HTN correlated with smoking; as the prevalence of HTN increased, the rate of smoking frequency increased. Choi et al. and Zhang et al. also reported results consistent with ours; people with prediabetes had a higher prevalence of HTN than those without diabetes undergoing elective CAG [12,13]. The reason for this explained by Kamalesh et al. in their study [14]. Patients with elevated HbA1c levels had a strong family history of CAD (P = .005) in both the prediabetes and diabetes groups compared to the non-diabetes group. Similarly, Acar et al. found that the prediabetes and diabetes groups had a higher prevalence of dyslipidemia based on a standard screen test [15]. Ashraf et al. and Ertan et al. evaluated the Gensini score and determined that the HbA1c level was linked with cardiac disease severity, demonstrating that HbA1c level and coronary heart disease (CHD) severity correlate [6,16]. Dong et al. enrolled 409 patients suspected of having a myocardial infarction undergoing CAG; they found that the severity of CHD and coronary stenosis increased with elevated HbA1c levels [17]. Another study by Muhammed et al. reported that prediabetic patients have similar CAD pattern and severity as that of diabetic patients, and have severe CAD severity and left main stem disease as compared to euglycemic patients [10]. These results are similar with our results as we also found a higher burden of triple valve disease, and double vessel disease in prediabetic and diabetic patients ([28.18%, 19.09%], [32.68%, 20.98%], versus [20.28%, 12.59%] in euglycemic patients, respectively [P = .01]). The Gensini score in our study was 28.0 ± 6.0 in prediabetic patients, higher than those of euglycemic and diabetic patients (F = 43.52, P < .001).

Prediabetic patients have more severe CAD when compared to euglycemic patients. So, recommending a healthy lifestyle, timely medical treatment, and regular physical activity may prevent prediabetes in those prone to diabetes and decrease their risk of cardiovascular disease.

A limitation of this research is that this was a single-center study with a limited sample size. Similar studies should be carried out in a larger population center with more variables to confirm the severity of CAD outcomes.

## Conclusions

The present study showed that coronary artery atherosclerotic disease increases parallel to HbA1c severity. Our study results showed a strong association of prediabetes with the severity of CAD. Therefore, the early identification of patients with prediabetes and implementation of vigorous lifestyle improvements and treatment modalities can help to convert those with prediabetes back to a state of euglycemia, helping to mitigate the burden of CAD in this population.

## References

[1] Tabák AG, Herder C, Rathmann W, Brunner EJ, Kivimäki M (2012). Prediabetes: a high-risk state for diabetes development. Lancet.

[2] Fredheim JM, Rollheim J, Omland T, Hofsø D, Røislien J, Vegsgaard K, Hjelmesæth J (2011). Type 2 diabetes and prediabetes are associated with obstructive sleep apnea in extremely obese subjects: a cross-sectional study. Cardiovasc Diabetol.

[3] Diabetes Prevention Program Research Group (2007). The prevalence of retinopathy in impaired glucose tolerance and recent-onset diabetes in the Diabetes Prevention Program. Diabet Med.

[4] Aamir AH, Ul-Haq Z, Mahar SA (2019). Diabetes Prevalence Survey of Pakistan (DPS-PAK): prevalence of type 2 diabetes mellitus and prediabetes using HbA1c: a population-based survey from Pakistan. BMJ Open.

[5] Færch K, Vistisen D, Johansen NB, Jørgensen ME (2014). Cardiovascular risk stratification and management in prediabetes. Curr Diab Rep.

[6] Ertan C, Ozeke O, Gul M (2014). Association of prediabetes with diffuse coronary narrowing and small-vessel disease. J Cardiol.

[7] Tomizawa N, Inoh S, Nojo T, Nakamura S (2016). The association of hemoglobin A1c and high risk plaque and plaque extent assessed by coronary computed tomography angiography. Int J Cardiovasc Imag.

[8] The International Expert Committee (2009). International Expert Committee report on the role of the A1C assay in the diagnosis of diabetes. Diabetes Care.

[9] American Diabetes Association (2016). Standards of medical care in diabetes—2016: summary of revisions. Diabetes Care.

[10] Muhammed A, Zaki MT, Elserafy AS, Amin SA (2019). Correlation between prediabetes and coronary artery disease severity in patients undergoing elective coronary angiography. Egypt Heart J.

[11] Kataoka Y, Yasuda S, Morii I, Otsuka Y, Kawamura A, Miyazaki S (2005). Quantitative coronary angiographic studies of patients with angina pectoris and impaired glucose tolerance. Diabetes Care.

[12] Choi WG, Rha S-W, Choi BG (2018). The impact of prediabetes on two-year clinical outcomes in patients undergoing elective percutaneous coronary intervention. Yonsei Med J.

[13] Zhang S, Dai J, Jia H (2018). Non-culprit plaque characteristics in acute coronary syndrome patients with raised hemoglobinA1c: an intravascular optical coherence tomography study. Cardiovasc Diabetol.

[14] Kamalesh M, Subramanian U, Sawada S, Eckert G, Temkit M, Tierney W (2006). Decreased survival in diabetic patients with heart failure due to systolic dysfunction. Eur J Heart Fail.

[15] Açar B, Ozeke O, Karakurt M (2019). Association of prediabetes with higher coronary atherosclerotic burden among patients with first diagnosed acute coronary syndrome. Angiology.

[16] Ashraf H, Boroumand MA, Amirzadegan A, Talesh SA, Davoodi G (2013). Hemoglobin A1C in non-diabetic patients: an independent predictor of coronary artery disease and its severity. Diabetes Res Clin Pract.

[17] Dong X, Zhou L, Zhai Y (2008). Impaired fasting glucose and the prevalence and severity of angiographic coronary artery disease in high-risk Chinese patients. Metabolism.

